# Designed Mini Protein 20 Mimicking Uricase Encapsulated in ZIF-8 as Nanozyme Biosensor for Uric Acid Detection

**DOI:** 10.3390/nano12132290

**Published:** 2022-07-04

**Authors:** Siti Fatimah Nur Abdul Aziz, Abu Bakar Salleh, Siti Efliza Ashari, Yahaya M. Normi, Nor Azah Yusof, Shahrul Ainliah Alang Ahmad

**Affiliations:** 1 Department of Chemistry, Faculty of Science, Universiti Putra Malaysia, Serdang 43400, Selangor, Malaysia; fatimahnur_siti@yahoo.com (S.F.N.A.A.); azahy@upm.edu.my (N.A.Y.); 2 Enzyme and Microbial Technology Research Centre (EMTech), Faculty of Biotechnology and Biomolecular Sciences, Universiti Putra Malaysia (UPM), Serdang 43400, Selangor, Malaysia; abubakar@upm.edu.my (A.B.S.); normi_yahaya@upm.edu.my (Y.M.N.); 3 Center of Foundation Studies for Agricultural Science, Universiti Putra Malaysia, Serdang 43400, Selangor, Malaysia; ct_efliza@upm.edu.my; 4 Institute of Nanoscience and Nanotechnology (ION2), Universiti Putra Malaysia, Serdang 43400, Selangor, Malaysia; 5 Department of Cell and Molecular Biology, Faculty of Biotechnology and Biomolecular Sciences, Universiti Putra Malaysia, Serdang 43400, Selangor, Malaysia

**Keywords:** uric acid, ZIFs, uricase, nanozymes, RSM

## Abstract

This work presents the use of encapsulated mini protein 20 mimicking uricase (mp20)-zeolitic imidazolate framework-8 (ZIF-8) as a bioreceptor for the development of a nanozyme-based electrochemical biosensor for uric acid detection. The electrochemical performance of the biofunctionalized mp20@ZIF-8 on the reduced graphene oxide/screen-printed carbon electrode (rGO/SPCE) was investigated by optimizing operating parameters such as pH, deposition potential, and deposition time using a central composite design-response surface methodology (CCD-RSM). The quadratic regression model was developed to correlate the combination of each variable to the oxidation current density as a response. A significant effect on current response was observed under optimized conditions of pH of 7.4 at −0.35 V deposition potential and 56.56 s deposition time, with *p* < 0.05 for each interacted factor. The obtained coefficient of determination (R^2^) value of 0.9992 indicated good agreement with the experimental finding. The developed nanozyme biosensor (mp20@ZIF-8/rGO/SPCE) exhibited high selectivity in the presence of the same fold concentration of interfering species with a detection limit of 0.27 μM, over a concentration range of 1 to 34 μM. The practicality of the tailored biosensor in monitoring uric acid in human serum and urine samples was validated with high-performance liquid chromatography (HPLC) and a commercial uric acid meter. Hence, nanozyme-based is a promising platform that offers a rapid, sensitive, selective, and low-cost biosensor for the non-enzymatic detection of uric acid in biological samples.

## 1. Introduction

Recent demand in the disease diagnosis field and treatment requires fast and efficient screening devices to detect and quantify metabolites produced from crucial biochemical processes that exist in human physiological fluids. Uric acid or urate (2,6,8-trihydroxypurine), one of the biomolecules, is an end product produced from the metabolization of purine alkaloids that can be found in urine, saliva, and serum [1,2]. The abnormal increase in serum uric acid (>300–500 µM) is associated with multiple health disorders such as gout, hyperuricemia, arthritis, Lesch-Nyhan syndrome, diabetes, metabolic syndrome, hypertension, renal and cardiovascular diseases due to excess deposition of urate crystals [2,3,4,5].

A conventional approach such as HPLC has been extensively used as a clinical method to recognize uric acid where uricase is employed to catalytically oxidize the urate, forming a soluble form of allantoin and hydrogen peroxide (H_2_O_2_) as a product. The amount of produced H_2_O_2_ is quantified via oxidation of the phenolic dye used in the protocol [1]. Similarly, with enzymatic test kits, chromatography and chemiluminecence techniques require tedious sample pre-treatment, long incubation times, expensive equipment and highly skilled personnel needs regardless of their high accuracy [5,6]. Owing to its simplicity, low cost, rapid and high specificity features, the electrochemical sensor has received a great deal of attention as a useful tool in monitoring uric acid [6,7]. The construction of an electrochemical biosensor is specifically portrayed as an alternative approach that can interact selectively with the utilized biointerface system. The employment of an enzyme for the recognition of uric acid could minimize the interference effects of substances such as dopamine, ascorbic acid, bilirubin, and nitrite, which regularly coexist in biological matrices [4]. Nevertheless, enzyme stability, high cost, and difficulty in storage have become the major limitations of its potential in constructing robust electrochemical interfaces.

Artificial enzymes (or nanozymes) based on nanoporous materials are predicted to become potential surrogates and competitors for natural enzymes in practical applications by minimizing these issues. Nanoporous metal-organic frameworks (MOFs) have fascinated scientific interest with their unique design, high surface area, and porosity with tunable pore size. Among its outstanding sub-classes, zeolitic imidazolate frameworks (ZIFs) have been reported to be one of the most effective exterior hosts for protecting a wide range of biomolecules against denaturation and degradation conditions due to their green synthesis steps, high thermal and chemical stability characteristics [8,9].

A developed analytical method needs to acquire maximum efficiency and performance for practical purposes. The response surface methodology (RSM) approach is one of the multivariate statistical techniques that have been extensively implemented to overcome classical optimization drawbacks such as time-consuming and lack of data evaluation as it involves a one-variable-at-a-time approach. Among the second-order symmetrical models applied, central composite design (CCD) is widely applied to estimate the parameters using the method of least squares and describe the behavior of the response in the selected design space [10,11]. An empirical model is created to predict the adequate experimental conditions, which are then able to reduce the number of experiments and produce a high impact response [12]. Based on the design expert, an experimental mathematical equation was also established to predict the correlation between the response and selected variables and optimize the multi-factor tests. Herein, this work is to investigate the influence of different electrochemical variables on the developed biosensor performance towards uric acid detection, and the determination of the optimal detection condition was investigated using three key parameters (pH, deposition potential, and deposition time) by utilizing the high accuracy and predictability design method.

In the present work, the designed bioinspired nanozyme mimicking uricase (mp20) is devoted to introducing strategic concepts in the development of electrochemical biosensors for uric acid recognition. Concisely, a miniaturized version of tetrametric uricase, namely mp20, has been designed and constructed specifically for the recognition of uric acid via a de novo approach by removing step-by-step procedures from the tetrameric native uricase (2yzb) of the *Arthrobacter globiformis* crystal structure to active sites that are located at the interface of monomer A and D. Monomer A-D was cut in half and left the active site and neighbor residues. However, it did not exhibit the observable catalytic activity of interest via enzymatic assay. Nevertheless, the binding of uric acid to the mini protein 20 was recorded via isothermal titration calorimetry and was postulated to occur through its contacting residue, which served as a binding site.

**Figure 1:**
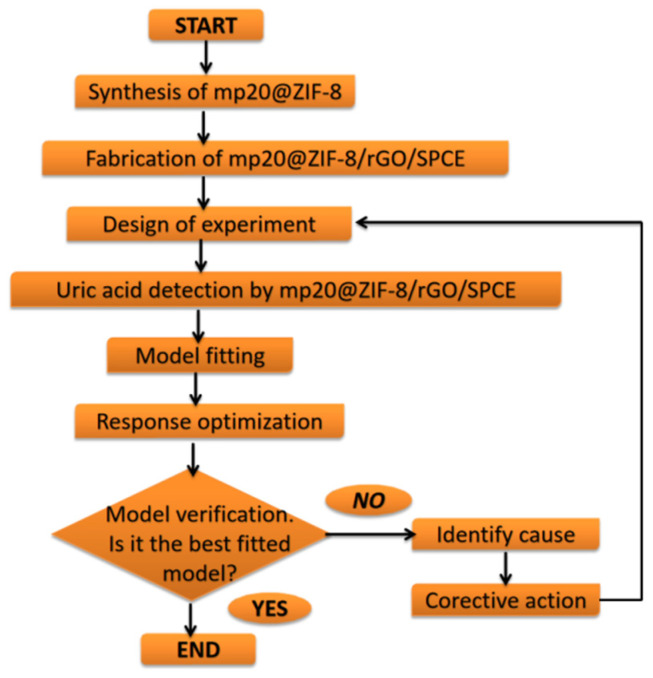
Flow chart of RSM optimization studies for uric acid detection based on mp20@ZIF-8/rGO/SPCE electrode.

The encapsulated nanozyme in ZIF-8 that is further modified on a reduced graphene oxide/screen-printed carbon electrode (rGO/SPCE) allows enhanced loading at the surfaces, resulting in improved electrochemical responses. The effects of operating parameters such as supporting electrolyte, pH, deposition potential, and deposition time were investigated and optimized through central composite design (CCD) combined with response surface methodology (RSM). The performance of the developed biosensor was compared. Figure 1 illustrates the designed process, including the synthesis of the mp20@ZIF-8 biocomposite and the subsequent optimization and validation of the model for the current response of the uric acid detection.

## 2. Experimental Section

### 2.1. Materials

All chemicals were analytical grade and were used without further purification. Glucose (C_6_H_12_O_6_), creatinine (C_4_H_7_N_3_O), and potassium chloride (KCl) were purchased from R&M. Zinc nitrate hexahydrate (Zn(NO_3_)_2_.6H_2_O, 99% purity), uric acid (C_5_H_4_N_4_O_3_, 99% purity) and L-cysteine (C_3_H_7_NO_2_S) were obtained from Sigma-Aldrich. Phosphate buffer saline solution (PBS, pH 7.4), potassium ferricyanide (K_3_[Fe(CN)]_6_) and potassium ferrocyanide (potassium hexacyanoferrate(II) *trihydrate*; K_4_[Fe(CN)]_6._3H_2_O) were obtained from Bendosen, while imidazole (C_3_H_4_N_2_), sodium chloride (NaCl), glycine, and sodium dodecyl sulfate (SDS) were supplied by Merck, Germany. Graphene oxide (GO), 2-methylimidazole (CH_3_C_3_H_2_N_2_H, Hmim, 99% purity), ascorbic acid (C_6_H_8_O_6_), urea (CH_4_N_2_O) were purchased separately from Advanced Solutions Sdn. Bhd., Acros Organics, and HmbG, respectively. Deionized water (18.2 MΩ·cm at 25 °C, Milli-Q) was used throughout the experiments. The design and construction of mini protein (mp20) mimicking uricase filed in PI2016700062, was used as a nanozyme. Screen-printed carbon electrodes (SPCEs), which consisted of a chemically inert electrode system with a 3 mm diameter of working, reference, and counter electrodes were printed and purchased from Rapid Labs Sdn. Bhd.

### 2.2. Instruments

Powder X-ray diffraction (PXRD) analysis was performed using a diffractometer (XRD-6000, Shimadzu, Kyoto, Japan) with a CuKα source, with a scan step of 0.05°, time per step of 20, and a scan range between 3° to 50°. The intensity of oxygen functionalities of GO and rGO on working electrodes was assessed by Raman spectroscopy using the Witec instrument model Alpha 300R. The surface morphologies of electrodes were observed with field emission scanning microscopy (FESEM) (Nova Nano SEM 230, FEI, Hillsboro, OR, USA). The electrochemical measurements by cyclic voltammetry (CV) and differential pulse voltammetry (DPV) were run using Autolab instrument Model Autolab Type III (Eco Chemie B. V., Utrecht, The Netherlands) and FRA impedance potentiometric module FRA32M with a wide frequency range of 10 µHz to 32 MHz for conducting electrochemical impedance spectroscopy (EIS) procedure. The data analyses were interpreted using Nova 1.11 software and charge transfer resistance (Rct) data of electrode surfaces concurrently was analyzed by NOVA 2.1 software, including the prediction of Randles equivalent model fitted circuit.

### 2.3. Methodology

#### 2.3.1. Synthesis of mp20@ZIF-8 Biocomposite

ZIF-8 and mp20@ZIF-8 were synthesized according to previously published protocol with slight modifications. Zn(NO_3_)_2_·6H_2_O (0.31 M, 250 μL) was added rapidly into solution of 2-methylimidazole (2.5 M, 2.5 mL) containing mini protein 20 mimicking uricase (mp20) (0.68 mg/mL, 250 μL). The molar ratio of Zn(NO_3_)_2_·6H_2_O to 2-methylimidazole was ~ 1:8. The synthesized product of the instantaneously formed milky solution was then collected by centrifugation at 10,000 rpm for 15 min, washed with deionized water three times, and air-dried for further characterizations. The biocomposite was stored at 4 °C until further use [13,14]. The synthesis of ZIF-8 was conducted under a similar condition with the absence of the mp20 solution.

#### 2.3.2. Functionalization of mp20@ZIF-8/rGO/SPCE Electrode

Initially, graphene oxide solution (2.5 mg/mL) was dispersed in 0.10 M phosphate buffer saline (PBS) at pH 7.4, followed by 5 min sonication to form desired 1 mg/mL concentration. To prepare the first layer of the modified surface, 5 μL of diluted GO was drop cast onto the SPCE working surface and dried at room temperature before electrochemically reducing using cyclic voltammetry within a potential range of −1.0 to 1.0 V at a scan rate of 50 mV/s for 10 cycles. The successful GO reduction process was indicated by the presence of a distinct cathodic peak at −0.5 V. Subsequently, 5 μL of mp20@ZIF-8 biocomposite solution was dropped onto the modified SPCE and allowed to dry at ambient temperature to form mp20@ZIF-8/rGO/SPCE surface.

#### 2.3.3. Experimental Design on the Optimization Condition of Uric Acid Detection Based on Response Surface Methodology (RSM)

A central composite design (CCD) was employed, allowing for an adequate estimation of the variable effects towards uric acid detection based on statistical analysis and a generated polynomial regression model. The CCD is defined as followed in Equation (1):

(1) $$Y=\beta_{0}+\sum_{i=1}^{k}\beta_{i}x_{i}+\sum_{i=1}^{k}\beta_{ii}x_{i}^{2}+\sum_{i=1}^{k-1}\;\sum_{i>j}^{k}\beta_{ij}x_{i}x_{j}\;$$

whereby *Y* represents the response (dependent variable); *β*_0_ is the constant coefficient; *β_i_*, *β_ii_*, *β_ij_* and *k* are coefficients for the respective effects of linear, quadratic, interactions parameter and variables number, while *x_i_* and *x_j_* are the factors (independent variables). pH, deposition potential, and deposition time were denoted as coded values (*x_i_* = A, *x_j_* = B, and *x_k_* = C), and are considered the crucial variables in this study. Thus, *k* takes a value of 3, which changes Equation (1) to Equation (2):

(2) $$Y=\beta_{0}+\beta_{1}x_{1}+\beta_{2}x_{2}+\beta_{3}x_{3}+\beta_{11}x_{1}^{2}+\beta_{22}x_{2}^{2}+\beta_{33}x_{3}^{2}+\beta_{12}x_{1}x_{2}+\;\beta_{13}x_{1}x_{3}+\beta_{23}x_{2}x_{3}+\beta_{112}x_{1}^{2}x_{2}+{\;\beta }_{113}x_{1}^{2}x_{3}+{\;\beta }_{122}x_{1}x_{2}^{2}+{\;\beta }_{123}x_{1}x_{2}x_{3}$$

A total of 18 experimental runs (Table S1) were suggested, including 8 factorial, 4 axial, and 6 center points using a statistical package (Design Expert 6.0, Stat Ease Inc., Minneapolis, MN, USA). The significance of the designed model was evaluated based on the F-value and a prob > F value generated by analyzing the analysis of variance (ANOVA), while the quality and precision is determined based on the comparison of the regression (R^2^), adjusted R^2^, predicted R^2^, and lack-of-fit values. Optimization of the electrochemical detection of uric acid was performed by utilizing the differential pulse voltammetry (DPV) technique in the presence of 25 μM of uric acid in 0.10 M PBS solution (pH 7.4).

#### 2.3.4. Verification of the Model

The designed model was assessed by validating the experimental process on the current response of uric acid detection. The predicted value developed by the model was based on the desirable optimum condition of selected variables using a response surface analysis. The current outputs of the five experiments were compared with the generated predicted value, and the residual standard error percentage (RSE) between both values was determined.

#### 2.3.5. Electrochemical Detection of Uric Acid by Differential Pulse Voltammetry (DPV) Analysis

The mp20@ZIF-8/rGO/SPCE electrodes were immersed in an electrochemical cell that contained spiked uric acid (1–34 μM) in 5 mL of 0.1 M phosphate buffer saline (PBS) solution. The pre-concentration of the analyte was achieved by setting the deposition potential at −0.35 V, deposition time of 56.56 s, and conditioning potential of 0 V, followed by conditioning time and equilibrium time at 5 s in a potential window of −0.2 to 0.8 V.

## 3. Results and Discussion

### 3.1. Powder X-ray Diffraction (PXRD) Analysis

**Figure 2:**
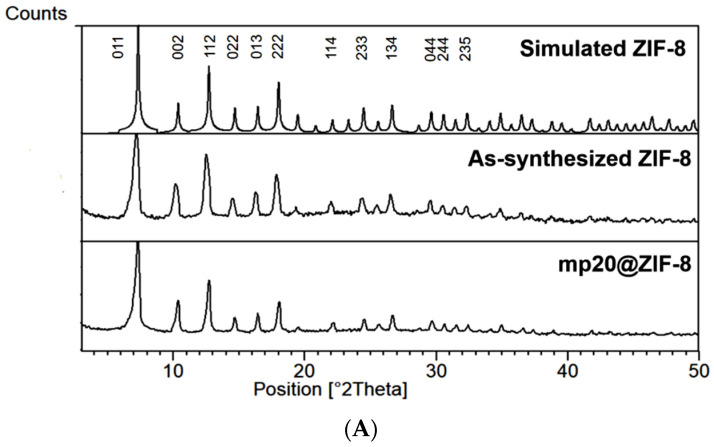

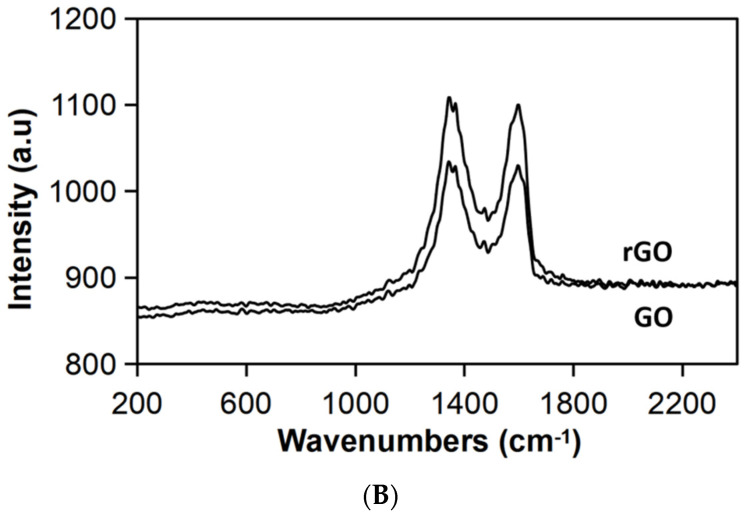
(**A**) PXRD patterns of ZIF-8 and mp20 encapsulated into ZIF-8 (mp20@ZIF-8) as compared to simulated pattern; (**B**) Raman spectra of GO and rGO on SPCE surface.

The crystallinity of as-synthesized ZIF-8 and mp20@ ZIF-8 was explored by performing powder X-ray diffraction (PXRD measurement (Figure 2A)). The emerging peaks at 2θ values appeared on the as-synthesized ZIF-8 that are consistent with those of simulated ZIF-8 crystal demonstrating a successful synthesis route [15,16]. As for mp20@ZIF-8, the similar prominent reflection of XRD patterns was also in congruence and well retained, indicating the successful formation of the ZIF-8 crystal phase with high framework stability and minimum impact on its crystallinity in presence of mp20.

### 3.2. Surface Analysis by Raman Spectroscopy and FESEM

**Figure 3:**
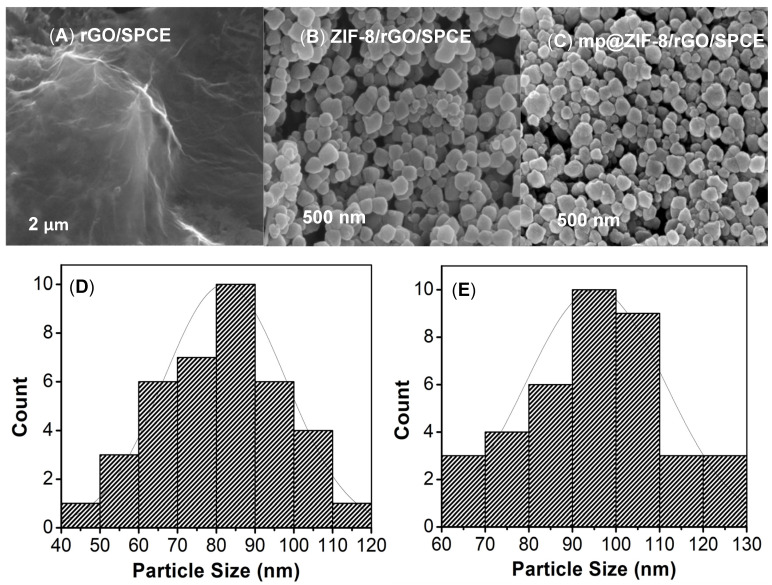
FESEM images display respective (**A**) rGO/SPCE, (**B**) ZIF-8/rGO/SPCE, and (**C**) mp20@ZIF-8/rGO/SPCE morphologies at 100kx magnification and histogram on particles size distribution of (**D**) ZIF-8/rGO/SPCE and (**E**) mp20@ZIF-8/rGO/SPCE.

The electrochemically reduced graphene oxide on the SPCE surface was characterized by Raman spectroscopy analysis. As seen in the spectra (Figure 2B), the intensity ratio of ID/IG at 1342 cm^−1^ and 1596 cm^−1^ was used to determine the degree of disorder, with a higher intensity ratio indicating a greater degree of disorder. Typically, the D band is ascribed to the out-of-plane breathing mode of sp^2^ carbon atoms because of defects, while the G band is assigned to the in-plane vibration of C atoms [17]. In comparison with the GO ratio (ID/IG: 1.004), a slightly higher ratio value of 1.009 was recorded for the electrochemically reduced GO surface. This is most likely due to a decrease in the average size of the sp^2^ domain and the removal of the oxygen groups from the GO interface, which is consistent with the literature [17,18]. The surface morphologies of modified electrodes were characterized by FESEM (Figure 3). As shown in Figure 3A, the FESEM image of electrochemically reduced graphene oxide reveals a crumpled, typical wrinkled, and wavy surface morphology, indicating a successful reduction of GO by an electrochemical technique, which is consistent with data published in the literature [19,20]. Figure 3B,C display the as-synthesized ZIF-8 and mp20@ZIF-8 modified on rGO, respectively. The images exhibit aggregated irregular spherical-like shapes with the particle sizes distribution particles (as shown in Figure 3D,E) of ZIF-8 were smaller (80–90 nm) than mp20@ZIF-8 (90–100 nm). The increase in size would likely indicate a fruitful in-situ biomimetic process. According to Liang et al., the formation of the biocomposite was priorly due to the binding of protein with metal ions or organic ligands to produce nanosize aggregates, which later induced the nucleation of ZIF crystals as outer protective shells and enhanced the robustness of the biomolecules [21]. This suggests that protein stabilization was aided by the coordination of Zn(II) ions with the protein’s amide bond and weak interactions likely hydrogen bonding and hydrophobic interactions of amino acids with the hydrophobic interior of the imidazole-containing building block of ZIFs [22,23].

### 3.3. Electrochemical Properties of Modified Electrodes

**Figure 4:**
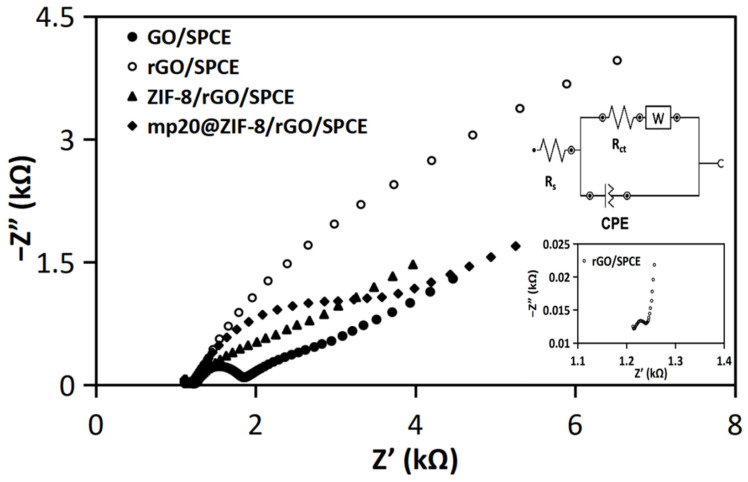
Nyquist plots of modified SPCEs in 5 mM Fe(CN)_6_^3−/4−^ in presence of 0.1 M KCl solution. The frequency range is 1 Hz to 100 kHz, and the amplitude is 0.2 V. The inset shows a magnification Nyquist plot of rGO/SPCE (below) and Randles equivalent circuit applied to fit the data (above).

The electrochemical properties of modified electrode interfaces were further evaluated using the electron impedance spectroscopy (EIS) technique. Figure 4 illustrates a Nyquist plot that exhibits the real and imaginary impedance (Z’ and −Z″) as components of the complex impedance (Z). In general, the semi-circular component at higher frequencies corresponds to the electron transfer process, whilst the linear section at lower frequencies corresponds to the diffusion process [24,25]. The Randles circuit was introduced to fit the impedimetric results shown in the inset of Figure 4 where charge transfer resistance (Rct) is defined as resistance related to dielectric and insulating characteristics at the electrode-electrolyte interface, Rs is the solution resistance, CPE is the constant phase element or Q, and Warburg (W) is the impedance due to mass transfer to the electrode interface observable at higher frequencies [24]. The diameter of the displayed semicircle corresponds to the electron transfer restricted process. The resistivity for each contact is equal to Rct [25,26]. The Rct value for each interface was ascertained to be 624.55 Ω, 89.34 Ω, 3402.8 Ω, and 3511 Ω for the corresponding GO/SPCE, rGO/SPCE, ZIF-8/rGO/SPCE, and mp20@ZIF-8/rGO/SPCE surfaces, respectively. The escalating resistivity values of the biofunctionalized electrode, as indicated by a larger semicircle, may be attributed to the insulator capabilities of ZIF-8 and the embedded biomolecule element. Impedimetric analysis of the rGO/SPCE surface revealed a straight-line segment, suggesting a highly rapid electron transfer rate.

### 3.4. Statistical Analysis of the Results and Model Fitting

Modeling based on the optimization of pH, deposition potential, and deposition time factors using RSM/CCD was conducted to maximize the current response based on uric acid detection by the mp20@ZIF-8/rGO/SPCE biosensor. The experimental data presented in Table S2 were analyzed and the significance of the model was determined based on a generated model F-value of 1108.75 with a prob > F value smaller than 0.0001 as shown in the ANOVA table. The values imply that the suggested model is significant, indicating that there is only a 0.01% chance that the large value of the model F-value occurs due to noise, while the small Prob > F value was less than 0.0500. Moreover, the model significance can be determined based on the tabulated lack-of-fit F-value of 1.52, which was greater than 0.1000. This indicates that the designed model possesses excellent predictability and has a negligible pure error. The experimental response based on the effect of experimental factors towards the current density of uric acid detection was modeled as the polynomial equation in coded factors (parameters) is shown in Equation (3):

(3) $$\begin{array}{c} \mathrm{Current}\;\mathrm{Density}=+5.50-0.22A-0.18B-0.33C-0.30A^{2}-0.31B^{2}-1.12C^{2}\\ \;-0.082\mathrm{AB}\;+0.47\mathrm{AC}-0.047\mathrm{BC} \end{array}$$

where A is pH, B is deposition potential and C is deposition time.

Based on the model fitted to the equation, the positive and negative signs of the term values imply synergistic and antagonistic effects, respectively. The positive correlation is assigned when there is a proportional increment between the values of the variables. Meanwhile, the negative correlation, which is represented by a negative sign, is assigned when the increment of one variable value is followed by decrements in the value of another variable, and vice versa. The terms A, B, C, A^2^, B^2^, C^2^, AB, AC, and BC summarized in Table S2 show significance between the selected independent variables towards current magnitude, based on the prob > F value which was less than 0.0500. Moreover, the comparison of the predicted R^2^ of 0.9904 is in reasonable agreement with the adjusted R^2^ of 0.9983, thus, suggesting the high precision of the fitted model in predicting the current response as displayed in Table 1. For an adequate and precise measurement of the signal-to-noise ratio, a ratio greater than 4 is desirable; hence, a ratio of 85.389 indicates that an adequate signal was obtained from this model. A standard deviation (S.D) value of 0.038, which was close to zero, and a small prediction error of sum of squares (PRESS) value of 0.14, both indicate a well-fitted model [27,28,29]. Additional predictions on the adequacy of the proposed model were determined using residual diagnostics. The data plot distribution is illustrated in Figure S1A, and indicates that the selected quadratic model was reasonable in assuming the variables’ responses to the experimental data. The predicted versus actual plot displays a good correlation between both responses, which fits with the linear regression by the R^2^ value of 0.9992. Moreover, the normal probability of the residual plots displayed in Figure S1B suggests that the errors are normally distributed, and were independent of homogeneous variance error, confirming the adequacy of the utilized model [30].

### 3.5. Effect of Each Factor

RSM employed with a CCD model is a straightforward method of validating the significance of the individual influences of A, B, and C variables towards their output. A prob > F value less than 0.0500 obtained from the ANOVA test represents the significance of each variable selected for this study. As can be seen in Figure S2A, the pH factor plot shows the highest current response was produced at pH 7.4 and decreases when approaching basic conditions at pH 9.4. At a lower pH, the electroactive species of uric acid tend to protonate, thus, resulting in better selectivity via molecular interactions with proton donating groups such as NH and OH. The trend illustrated in Figure S2B shows a significant individual effect of the deposition potential variable. The increasing potential for negative values result in a greater peak current reading than a positive potential. The uric acid likely underwent oxidation by releasing electrons into the solution prior to the reduction process on the working electrode surface. The progressive decrease in the current response at a positive potential was comparable with a previous study reported by Babaei, et al. [31]. Figure S2C demonstrates the effects of deposition time. The best peak current was acquired at 120 s of deposition time. Saturation of the analyte at the active site of modifier mp20@ZIF-8 possibly resulted in a lower current reading at a longer deposition time. Based on the plot trends, the interpretation of the three respective factors as significant independent variables agreed with the prob > F value.

### 3.6. Effect of Interaction between Factors

The interactive effects of independent variables on responses are visualized as 3D surface and 2D estimated contour plots using the fitted model equation and shown in Figure S3. The response surface plots for the peak current magnitude of uric acid detection as the output are displayed based on the interaction between two factors while one factor remains constant. The intense curvature and strong interaction between each factor indicate that the variables pH, deposition potential, and deposition time significantly affect the output current response.

### 3.7. Validation of the Predictive Model and Optimization of the Current Response

The validity of the experimental results was determined based on the accuracy of the generated value, which agrees with the theoretical and ultimately confirms the reliability of the experimental results. The desired parameters for the optimization of uric acid detection are tabulated in Table S3. Constraint selection was applied to produce a maximum peak current value using the point prediction option in the Design Expert software. Validation of the model was performed by conducting five different experiments. The outcome of the peak current responses was compared with the generated value from the model (Table 2). The percentage of residual standard error (RSE) between predicted and experimental current output was determined using Equation (4):

(4) $$\mathrm{Residual}\;\mathrm{standard}\;\mathrm{error}\;(\mathrm{RSE})\%=\frac{\mathrm{Experimental}\;\mathrm{value}-\mathrm{Predicted}\;\mathrm{value}}{\mathrm{Predicted}\;\mathrm{value}}\times 100\%$$

RSE percentage less than 2% indicates that precise data was obtained compared to the predicted one. Run number 1, which was the optimal condition predicted by the model based on the desired condition, was then used for the next experimental step.

### 3.8. Comparative Study and Analytical Performance of mp20@ZIF-8/rGO/SPCE Electrode towards Uric Acid

Figure S4 portrays a histogram of the current response of each modified electrode on SPCE in 1 μM uric acid. The interface of rGO/SPCE exhibited a higher current density for uric acid detection, possibly due to the increased surface area with excellent catalytic activity and the stronger hydrogen-bond acceptor strength of uric acid’s amide group, in contrast to GO/SPCE, which primarily involved weak-stacking with the electroactive species [32]. Peak current values were observed to be greater for ZIF-8/rGO/SPCE, most likely due to the synergistic effect of the conductive rGO and the larger surface area of porous ZIF-8, resulting in the efficient and sensitive detection of the target analyte [32]. An attempt to construct a biosensor for uric acid was made using mp20 modified on the surface of rGO. In comparison to ZIF-8/rGO, the oxidation peak of UA on mp20/rGO/SPCE was significantly reduced, indicating low catalytic activity of mp20/rGO/SPCE toward UA. This was consistent with the observation of mp20 in the uricase assay, which revealed that no activity was observed [33]. A significant improvement in current signal quality over other modified electrodes has been attained by encapsulating mp20 onto ZIF-8 and making modifications to its rGO/SPCE. This is attributable to the oxidation reaction of uric acid bound to active sites through substrate-enzyme interaction. Uric acid binding to ZIF-8 in mp20-encapsulated ZIF-8 could be ascribable to the biomimetic mineralization process, which is closely correlated to the conformation of biomolecules.

**Figure 5:**
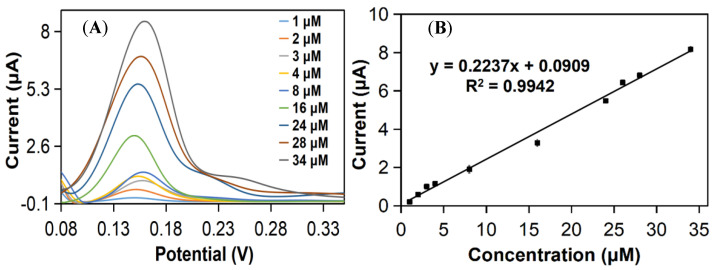
(**A**) DPV of the mp20@ZIF-8/rGO/SPCE after spiking of different uric acid concentrations in 0.1 M PBS at pH 7.4, and (**B**) calibration curve of current density as a function of the UA concentration.

**Figure 6:**
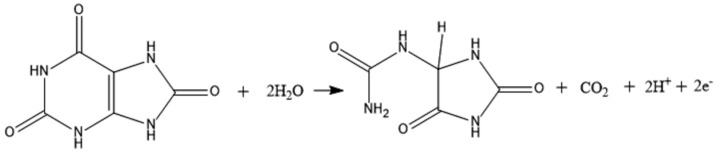
The proposed electrochemical oxidation of uric acid.

The developed mp20@ZIF-8/rGO/SPCE sensor’s sensitivity to uric acid was determined. Figure 5A depicts the DPV responses to several uric acid concentration additions. A calibration curve was constructed by plotting a linear relationship between the oxidation peak current and the concentration in the range of 1–34 μM (Figure 5B). The regression equation can be expressed as Ipa (μA) = 0.2237 (μM) + 0.909 (R^2^ = 0.9942) with a detection limit of 0.27 μM and a quantification limit of 0.91 μM (S/N = 3) using 3/s and 10/s, respectively, where σ and s represent the standard deviation of the blank and the slope of the fitted calibration curve, respectively. As shown in Table 3, the suggested sensor’s analytical performance in terms of LOD was compared to that of other previously published uric acid sensors, with the constructed sensor exhibiting promising analytical performance in comparison to previously established uric acid sensors. The endeavor to develop a biosensor for uric acid detection using mini protein mimics (mp20) as a replacement for native enzymes has shown great sensitivity and selectivity towards the target. This mini protein was developed with two active sites, Thr18 and Gln19, which are responsible for interactions with the substrate, uric acid (Figure S5) [33]. In contrast, the catalytic reaction of active uricase has resulted in the formation of H_2_O_2_. The electrochemical oxidation of uric acid by mp20@ZIF-8 on the rGO/SPCE biosensor involves the loss of two electrons and two protons, resulting in the formation of unstable bisdimine, which later hydrolyzes to uric acid-4,5 diol and decomposes to allantoin and CO_2_ on the working electrode. The electrochemical oxidation of uric acid on the modified electrode is depicted in Figure 6 [32,34,35]. The applicability of the developed biosensor for the detection of various uric acid concentrations was also evaluated using cyclic voltammetry (CV). As displayed in Figure S6, in the absence of uric acid, no peak was observed in the voltammogram. The addition of different concentrations of uric acid resulted in an irreversible peak exhibited at a potential of 0.11 V, which increases linearly with increasing uric acid concentration in the range of 40–140 μM. This demonstrates the targeted analyte could be detected well by the developed mp20@ZIF-8/rGO/SPCE simply via enzyme-substrate interaction. This confirms the favorable oxidation process of uric acid as proposed in the mechanism.

### 3.9. Selectivity, Reproducibility, and Stability Studies

To evaluate the selectivity of the developed biosensor towards uric acid determination, interference studies with some potential interfering compounds such as ascorbic acid, urea, glucose, and L-cysteine were conducted. There were no significant current changes in the presence of 22 μM of interfering compounds as shown in Table 4. This suggests that the fabricated sensor exhibited high selectivity towards uric acid with RSD values less than 5%, indicating an insignificant impact of interferents. The reproducibility and stability of the mp20@ZIF-8/rGO/SPCE sensor were also determined by calculating the RSD value of five successive modified electrodes under optimal experimental conditions. A value of 3.17% was recorded, suggesting excellent reproducibility (Figure S7). Additionally, the stability of the fabricated sensor was investigated by storing them in a refrigerator at 4 °C for six months. The peak density value decreased by approximately 8% from its initial current reading (RSD = 2.5%, N = 3), indicating good stability of the developed sensor.

### 3.10. Real Physiological Sample Analysis

**Figure 7:**
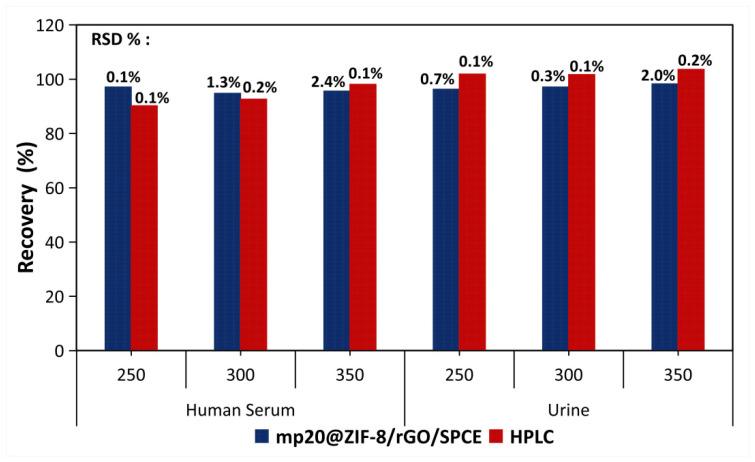
Data validation of uric acid in physiological fluids.

**Figure 8:**
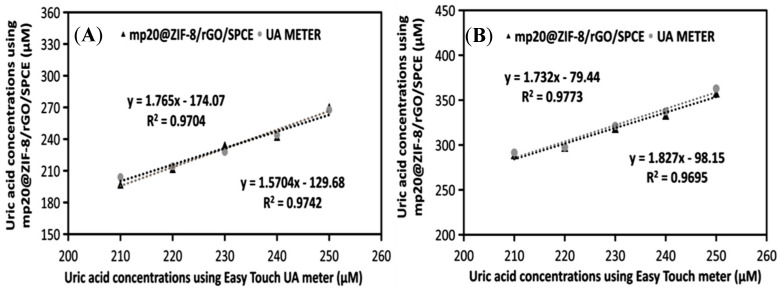
Data validation of uric acid by biosensor mp20@ZIF-8/rGO/SPCE and UA meter in physiological fluids; (**A**) human serum and (**B**) urine.

To ascertain the validity of the results and evaluate the practicality of the fabricated mp20@ZIF-8/rGO/SPCE biosensor, the concentrations of uric acid in urine and human serum samples were determined using the standard addition method. Human serum was centrifuged at 12,500 rpm for 15 min and diluted 100 times with 0.1 M PBS solution at pH 7.4. The same fold dilution was also performed with fresh urine samples to reduce the matrix effect of real samples without other pre-treatments [42]. The recovery of the spiked samples was found to be comparable with that of conventional HPLC techniques and commercialized EasyTouch meter, as shown in Figure 7 and Figure 8, respectively. The LOD of the proposed sensor for human serum and urine was calculated to be 0.478 μM and 0.487 μM, respectively. These LOD data show minimal differences between LOD in PBS buffer (0.27 μM), demonstrating that the developed sensor (mp20@ZIF-8/rGO/SPCE) is capable of detecting uric acid in a quantitative and specific manner in human serum and urine samples as well as in PBS.

## 4. Conclusions

The fabrication of the mp20@ZIF-8/rGO/SPCE biosensor was successfully optimized with the utilization of a three-factor of Central Composite Design response experimental design combining RSM in reaching optimal current density. The statistical analysis of the data indicated pH, deposition potential and deposition time parameters had a significant effect on the current response (*p* < 0.05). The adequacy of the model was proven as the actual values were in good agreement with the predicted values and the calculated RSE was less than 2%. Satisfactory analytical performance with an LOD of 0.27 μM and promising recovery percentages in biofluids suggest great practicality of the nanonzyme-based biosensor for the development of self-monitoring devices and clinical applications.

## Figures and Tables

**Table 1:** Statistical parameter of the model equation as obtained from ANOVA models.

Variables	Value
Standard Deviation (SD)	0.038
Mean	4.57
Coefficient of Variation (CV)	0.84
Predicted Residual Error Sum of Squares (PRESS)	0.14
R^2^	0.9992
Adjusted R^2^	0.9983
Predicted R^2^	0.9904
Adequate Precision	85.389

**Table 2:** Predicted and observed response values performed at optimum conditions of uric acid detection by mp20@ZIF-8/rGO/SPCE.

Number	pH	Deposition Potential, V	Deposition Time, s	Current, μA		RSE (%)
				Predicted	Experiment	
1	7.4	−0.35	56.56	5.20	5.19	0.19
2	7.4	−0.3	50.56	5.09	5.00	1.76
3	7.4	−0.4	46.56	5.10	5.08	0.39
4	7.4	−0.2	66.56	4.88	4.90	0.41
5	7.4	−0.45	40.56	4.85	4.86	0.21

**Table 3:** A comparison of different electrochemical biosensors for the detection of uric acid.

Fabricated Sensor	Linear Range (μM)	Detection Limit (μM)	Ref.
UOX-HRP, entrapment, carbon paste covered with poly(o-aminophenol)	Up to 100	3.0	[36]
UOX, glutaraldehyte cross-linking, polypyrrole membrane	1.0–50	0.5	[37]
uricase onto GO	20–490	3.45	[38]
immobilizing uricase in crosslinked chitosan network through glutaraldehyde, onto PBNPs/c-MWCNT/PANI/Au modified electrode	5–800	5.0	[39]
UOx/EDC:NHS/CZTS/ITO- bioelectrode	50–700	0.066	[40]
Uricase-overproducing strains of *Hansenula polymorpha* on graphite electrode	Up to 180	8.0	[41]
mp20 encapsulated ZIF-8 on reduced graphene oxide (mp20@ZIF-8/rGO/SPCE) electrode	1–34	0.27	present work

**Table 4:** Representative data of magnitude current changes during uric acid detection with the presence of several interferents at the same fold concentration (22 μM) in 0.1 M PBS solution at pH 7.4 and the mp20@ZIF-8/rGO/SPCE biosensor stability over time.

	Signal Changed (%)	RSD (%)
**Interferent**		
Ascorbic Acid	1.73	0.83
Urea	3.56	0.75
Glucose	3.08	2.68
L-Cysteine	2.70	3.46
Creatinine	0.71	1.64
**Stability (Days)**		
14	4	1.0
30	7	2.5
60	7	0.1
180	8	1.08

## Data Availability

The data in this study are available in this manuscript and supplementary material.

## Supplementary Materials

The following supporting information can be downloaded at: https://www.mdpi.com/article/10.3390/nano12132290/s1, Figure S1: (A) Plot of the predicted versus actual correlation (B) Plot of normal probability versus residuals of peak current magnitude; Figure S2: One factor plot of peak current magnitude versus (A) pH, (B) deposition potential, and (C) deposition time, Figure S3: Plot of 3D surface versus (A) pH, (B) deposition potential, and (C) deposition time; Figure S4: Bar chart of comparative study of different modified electrode; Figure S5: Diagram illustrates predicted interaction between mp20 with uric acid; Figure S6: Plot of CV curves of uric acid detection current versus potential; Figure S7: Graph of uric acid detection current versus number of electrode; Table S1: Table of central composite design (CCD) for the optimization of uric acid detection and the current response; Table S2: ANOVA table; Table S3: Table of constraints applied for the optimization.
