# Supplementary material for: Designed Mini Protein 20 Mimicking Uricase Encapsulated in ZIF-8 as Nanozyme Biosensor for Uric Acid Detection
Source: Nanomaterials (Basel). 2022 Jul 4;12(13):2290. doi: 10.3390/nano12132290 (PMC9267977; doi:10.3390/nano12132290)
Supplement: Supplementary file 1 [file nanomaterials-12-02290-s001.zip › nanomaterials-1787296-supplementary.pdf]

Supplementary Materials

Designed Mini Protein 20 Mimicking Uricase Encapsulated in ZIF-8 as Nanozyme Biosensor for Uric Acid Detection

Siti Fatimah Nur Abdul Aziz [1], Abu Bakar Salleh [2], Siti Efliza Ashari [3], Yahaya M. Normi [2,4,5], Nor Azah Yusof [1,4] and Shahrul Ainliah Alang Ahmad [1,4,*]

[1] Department of Chemistry, Faculty of Science, Universiti Putra Malaysia, Serdang 43400, Selangor, Malaysia; fatimahnur_siti@yahoo.com (S.F.N.A.A.); azahy@upm.edu.my (N.A.Y.)
[2] Enzyme and Microbial Technology Research Centre (EMTech), Faculty of Biotechnology and Biomolecular Sciences, Universiti Putra Malaysia (UPM), Serdang 43400, Selangor, Malaysia; abubakar@upm.edu.my (A.B.S.); normi_yahaya@upm.edu.my (Y.M.N.)
[3] Center of Foundation Studies for Agricultural Science, Universiti Putra Malaysia, Serdang 43400, Selangor, Malaysia; ct_efliza@upm.edu.my
[4] Institute of Nanoscience and Nanotechnology (ION2), Universiti Putra Malaysia, 43400 Serdang, 43400, Selangor, Malaysia
[5] Department of Cell and Molecular Biology, Faculty of Biotechnology and Biomolecular Sciences, Universiti Putra Malaysia, Serdang 43400, Selangor, Malaysia
* Correspondence: ainliah@upm.edu.my

Table S1. Central composite design (CCD) for the optimization of uric acid detection by mp20@ZIF-8/rGO/SPCE using the RSM method and results of the current response.

Standard	A: pH	B: Deposition Potential (V)	C: Deposition Time (s)	Peak Current (µA)	
				Experimental	Predicted
**1**	5.4	-0.50	30.00	4.87	4.84
**2**	9.4	-0.50	30.00	3.62	3.63
**3**	5.4	-0.10	30.00	4.72	4.74
**4**	9.4	-0.10	30.00	3.22	3.20
**5**	5.4	-0.50	210.00	3.32	3.34
**6**	9.4	-0.50	210.00	4.01	3.99
**7**	5.4	-0.10	210.00	3.06	3.05
**8**	9.4	-0.10	210.00	3.34	3.37
**11**	7.4	-0.50	120.00	5.34	5.37
**12**	7.4	-0.10	120.00	5.04	5.01
**13**	7.4	-0.30	30.00	4.70	4.71
**14**	7.4	-0.30	210.00	4.06	4.05
**15**	7.4	-0.30	120.00	5.51	5.50
**16**	7.4	-0.30	120.00	5.44	5.50
**17**	7.4	-0.30	120.00	5.48	5.50
**18**	7.4	-0.30	120.00	5.52	5.50
**19**	7.4	-0.30	120.00	5.54	5.50
**20**	7.4	-0.30	120.00	5.51	5.50

Table S2. ANOVA table of the response surface quadratic model.

Source	Sum of Squares	Degree of Freedom	Mean Square	F - value	Prob > F	Significant
**Model**	14.78	9	1.64	1108.75	< 0.0001	Significant
**A**	0.40	1	0.40	267.38	< 0.0001	Significant
**B**	0.32	1	0.32	213.90	< 0.0001	Significant
**C**	1.12	1	1.12	753.12	< 0.0001	Significant
**A^2**	0.070	1	0.070	47.04	0.0001	Significant
**B^2**	0.14	1	0.14	97.32	< 0.0001	Significant
**C^2**	1.88	1	1.88	1270.28	< 0.0001	Significant
**AB**	0.054	1	0.054	36.76	0.0003	Significant
**AC**	1.73	1	1.73	1167.80	< 0.0001	Significant
**BC**	0.018	1	0.018	12.19	0.0082	Significant
**Residual**	0.012	8	0.00148			
**Lack of Fit**	0.00565	3	0.00188	1.52	0.3180	Not significant
**Pure Error**	0.00620	5	0.00124			
**Corrected Total**	14.79	17				

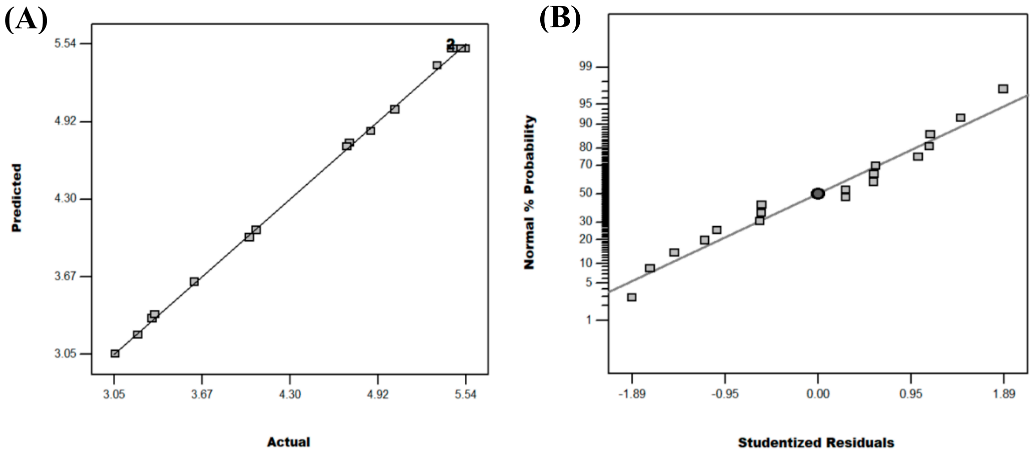

Figure S1. (A) Plot of the predicted versus actual correlation of the two values and (B) Normal probability plot of the residual for peak current magnitude of uric acid detection by mp20@ZIF-8/rGO/SPCE.

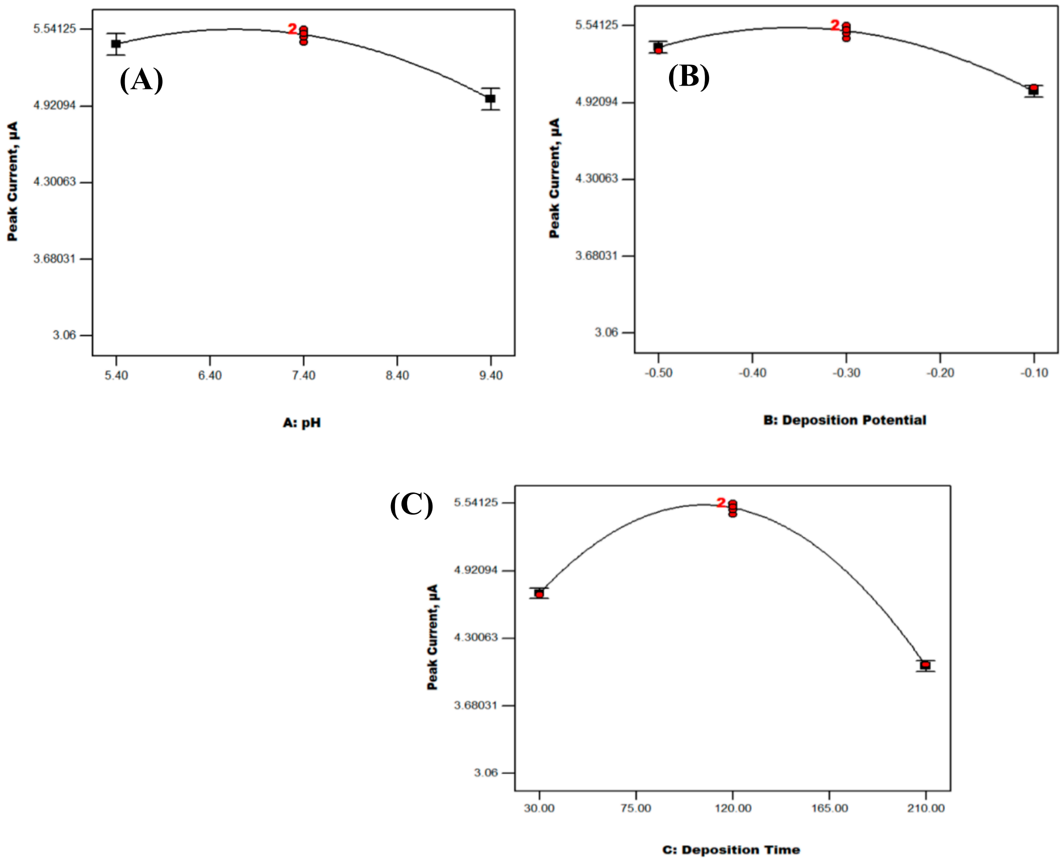

Figure S2. One factor plot of peak current magnitude as a function of (A) pH (at deposition potential = –0.3 V; deposition potential = 120 s); (B) deposition potential of uric acid (pH = 7.4; deposition time = 120 s); and (C) deposition time of uric acid (pH = 7.4; deposition potential = –0.30 V).

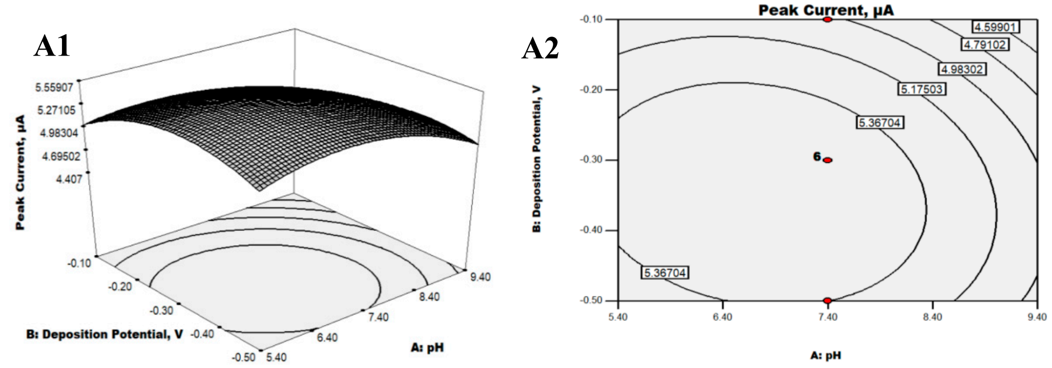

(A)

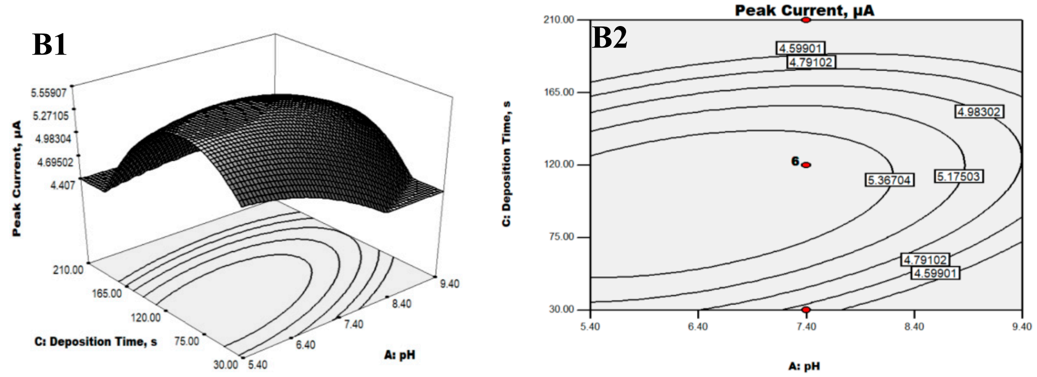

(B)

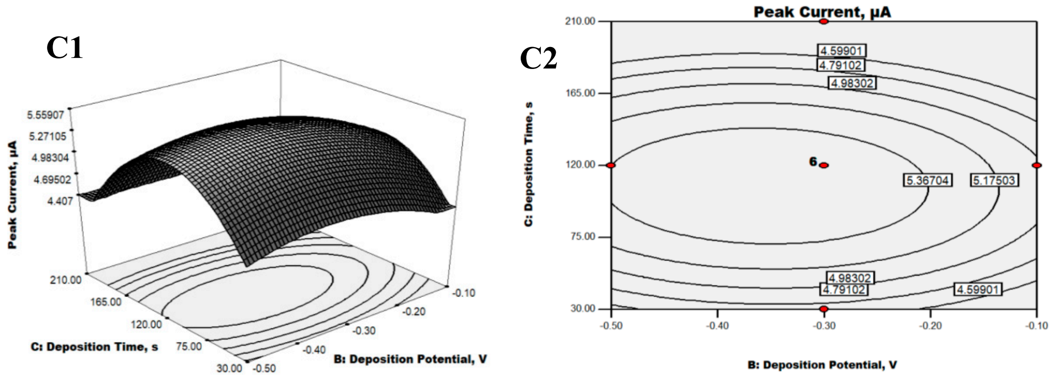

(C)

Figure S3. 3D surface and 2D contour plots of peak current as a function of (A) pH and deposition potential, (B) pH and deposition time, and (C) deposition potential and deposition time.

Table S3. Constraints applied for the optimization of the peak current of uric acid detection by mp20@ZIF-8/rGO/SPCE

**Variables**	**Goal**	**Limit**	
		**Lower**	**Upper**
**pH**	is target = 7.4	5.4	9.4
**Deposition Potential , V**	is in range	–0.50	–0.10
**Deposition Time, s**	minimize	30.00	210.00
**Peak Current, µA**	maximize	3.06	5.54

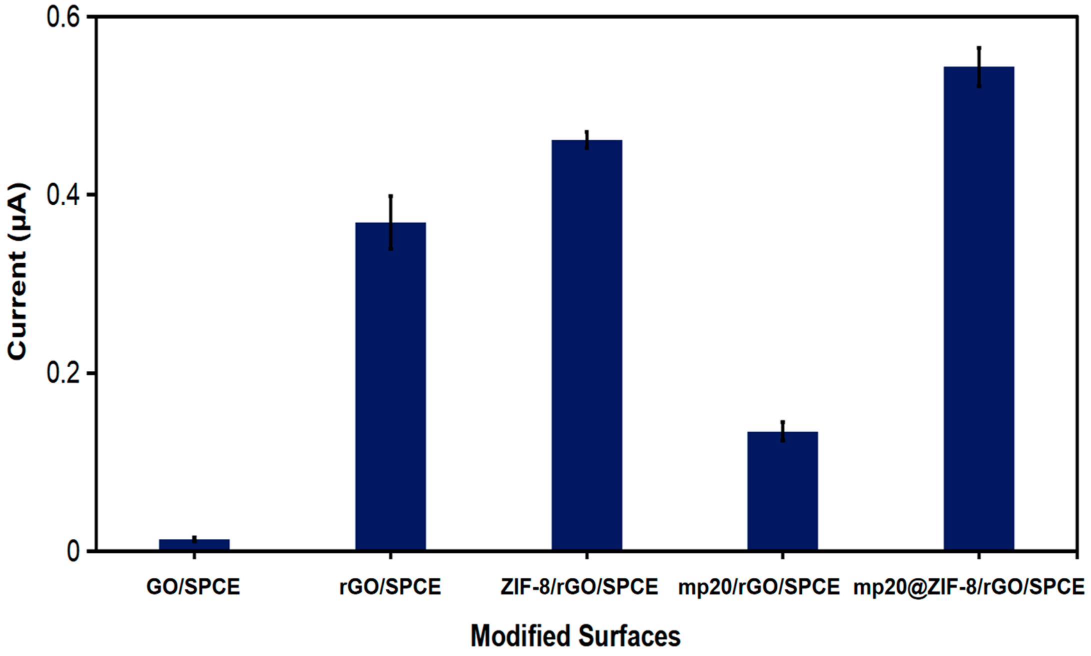

Figure S4. Comparative study of different modified surfaces towards the detection of 1 µM uric acid in 0.1 M PBS solution at pH 7.4.

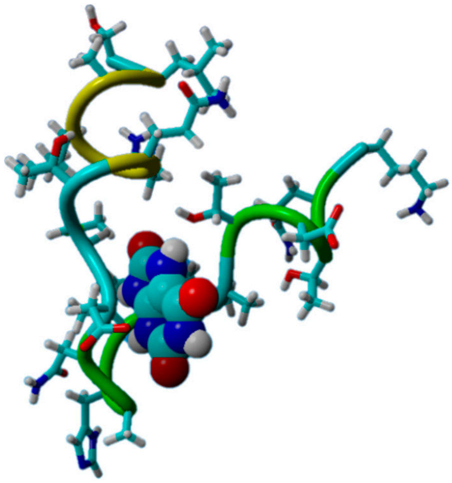

Peptide	*Contacting Residue*
mp20	Val13,Val14,Ala15**, Thr18, Gln19**

Figure S5. Predicted interaction between mp20 with target analyte uric acid (colored balls) and contacting residues of peptide involved. Residues that form hydrogen bonds with uric acid are indicated in bold.

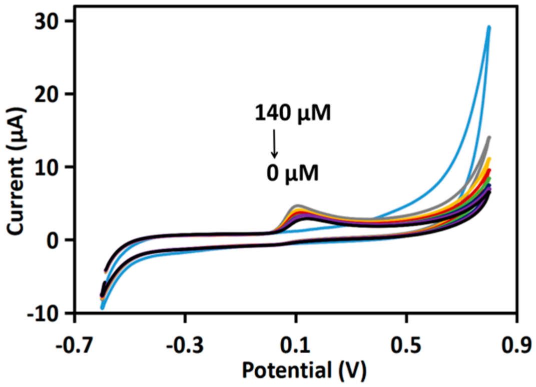

Figure S6. CV curves of the mp20@ZIF-8/rGO/SPCE biosensor after spiking of different uric acid concentrations in 0.1 M PBS (pH 7.4) at scan rate of 100 mV/s.

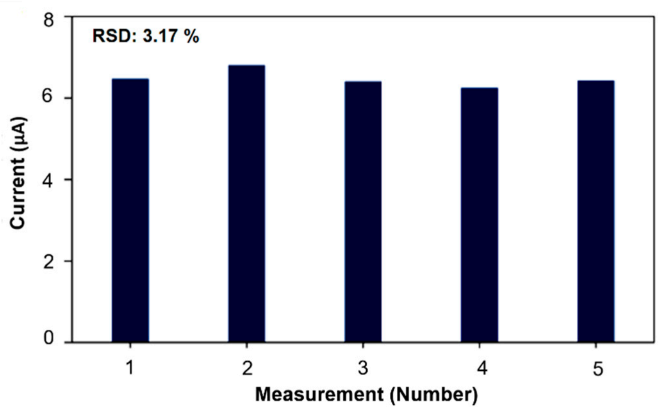

Figure S7. The reproducibility study of mp20@ZIF-8/rGO/SPCE in the detection of 26 μM uric acid.
